# Supplementary material for: Identification and evaluation of the novel genes for transcript normalization during female gametophyte development in sugarcane
Source: PeerJ. 2021 Oct 19;9:e12298. doi: 10.7717/peerj.12298 (PMC8532975; doi:10.7717/peerj.12298)
Supplement: Supplemental Information 1 [file peerj-09-12298-s001.docx]

**Table S1.** The top 50 stably expressed genes for different developmental stages of sugarcane female gametophyte (The FPKM values of the top 50 genes).

| **Gene ID** | **AC** | **MMC** | **Meiosis** | **Mitosis** | **Mature** | **Mean** | **SD** | **CV** |
| --- | --- | --- | --- | --- | --- | --- | --- | --- |
| Sspon.03G0028120 | 14.42 | 15.98 | 16.74 | 16.62 | 16.02 | 15.95 | 0.92 | 0.06 |
| Sspon.01G0006710 | 102.71 | 89.00 | 96.34 | 88.62 | 101.01 | 95.54 | 6.57 | 0.07 |
| Sspon.08G0004380 | 26.80 | 26.91 | 29.62 | 27.07 | 24.24 | 26.93 | 1.91 | 0.07 |
| Sspon.01G0037150 | 141.36 | 157.32 | 165.67 | 166.63 | 140.96 | 154.39 | 12.61 | 0.08 |
| Sspon.06G0026890 | 49.89 | 53.20 | 42.60 | 46.60 | 47.07 | 47.87 | 3.96 | 0.08 |
| Sspon.08G0021360 | 16.33 | 15.74 | 17.63 | 14.93 | 18.54 | 16.64 | 1.45 | 0.09 |
| Sspon.01G0020070 | 27.09 | 26.97 | 21.79 | 24.98 | 23.59 | 24.88 | 2.26 | 0.09 |
| Sspon.03G0026510 | 29.58 | 25.08 | 24.55 | 28.27 | 24.29 | 26.35 | 2.41 | 0.09 |
| Sspon.03G0047080 | 11.25 | 12.26 | 10.91 | 9.42 | 10.98 | 10.96 | 1.02 | 0.09 |
| Sspon.03G0004010 | 32.30 | 26.04 | 27.43 | 25.85 | 27.57 | 27.84 | 2.61 | 0.09 |
| Sspon.06G0031000 | 12.60 | 9.93 | 11.74 | 12.68 | 11.52 | 11.69 | 1.11 | 0.09 |
| Sspon.01G0010170 | 36.44 | 40.11 | 33.17 | 37.41 | 30.22 | 35.47 | 3.84 | 0.11 |
| Sspon.01G0039090 | 28.81 | 26.91 | 33.39 | 25.21 | 27.36 | 28.34 | 3.10 | 0.11 |
| Sspon.04G0009400 | 15.74 | 12.24 | 13.62 | 14.66 | 16.30 | 14.51 | 1.64 | 0.11 |
| Sspon.01G0047550 | 48.49 | 44.84 | 35.22 | 40.46 | 39.83 | 41.77 | 5.07 | 0.12 |
| Sspon.04G0012190 | 69.92 | 66.48 | 53.77 | 63.66 | 53.43 | 61.45 | 7.51 | 0.12 |
| Sspon.08G0013450 | 5.80 | 6.43 | 5.93 | 6.40 | 7.84 | 6.48 | 0.81 | 0.13 |
| Sspon.01G0002680 | 23.08 | 18.41 | 22.44 | 23.19 | 17.73 | 20.97 | 2.67 | 0.13 |
| Sspon.01G0060460 | 4.10 | 3.41 | 3.57 | 3.09 | 2.93 | 3.42 | 0.46 | 0.13 |
| Sspon.04G0015990 | 38.54 | 45.84 | 40.92 | 54.04 | 42.31 | 44.33 | 6.04 | 0.14 |
| Sspon.05G0016540 | 8.78 | 10.11 | 9.77 | 9.10 | 6.89 | 8.93 | 1.25 | 0.14 |
| Sspon.03G0006680 | 14.69 | 19.98 | 17.55 | 20.34 | 15.84 | 17.68 | 2.49 | 0.14 |
| Sspon.01G0028420 | 14.66 | 13.15 | 11.43 | 13.85 | 10.25 | 12.67 | 1.80 | 0.14 |
| Sspon.02G0034560 | 77.31 | 76.15 | 100.34 | 70.52 | 87.37 | 82.34 | 11.75 | 0.14 |
| Sspon.04G0004910 | 81.46 | 94.69 | 76.15 | 88.38 | 109.30 | 90.00 | 12.86 | 0.14 |
| Sspon.02G0032880 | 18.16 | 23.68 | 23.09 | 27.53 | 24.73 | 23.44 | 3.41 | 0.15 |
| Sspon.01G0058250 | 8.58 | 7.43 | 10.76 | 10.54 | 9.24 | 9.31 | 1.39 | 0.15 |
| Sspon.02G0019370 | 40.96 | 38.76 | 30.39 | 32.11 | 29.69 | 34.38 | 5.14 | 0.15 |
| Sspon.02G0036810 | 12.70 | 13.24 | 12.12 | 11.16 | 8.80 | 11.60 | 1.74 | 0.15 |
| Sspon.03G0011200 | 55.93 | 57.27 | 60.51 | 78.76 | 58.25 | 62.14 | 9.44 | 0.15 |
| Sspon.01G0058290 | 2.65 | 2.99 | 2.20 | 2.82 | 2.09 | 2.55 | 0.39 | 0.15 |
| Sspon.07G0006260 | 61.00 | 58.73 | 67.38 | 78.47 | 52.61 | 63.64 | 9.83 | 0.15 |
| Sspon.03G0031670 | 12.13 | 11.71 | 10.83 | 12.75 | 8.31 | 11.15 | 1.73 | 0.16 |
| Sspon.02G0024360 | 15.11 | 13.01 | 19.93 | 17.25 | 16.42 | 16.34 | 2.56 | 0.16 |
| Sspon.04G0027790 | 158.54 | 194.95 | 243.50 | 226.53 | 206.44 | 205.99 | 32.41 | 0.16 |
| Sspon.08G0006390 | 56.59 | 53.24 | 52.00 | 36.63 | 54.35 | 50.56 | 7.97 | 0.16 |
| Sspon.01G0008680 | 8.64 | 6.97 | 6.75 | 5.66 | 7.86 | 7.17 | 1.13 | 0.16 |
| Sspon.01G0037490 | 11.08 | 9.90 | 12.20 | 8.05 | 11.82 | 10.61 | 1.68 | 0.16 |
| Sspon.01G0026700 | 55.06 | 56.64 | 60.75 | 79.58 | 59.54 | 62.32 | 9.91 | 0.16 |
| Sspon.01G0061760 | 53.25 | 48.68 | 45.83 | 36.54 | 38.02 | 44.46 | 7.09 | 0.16 |
| Sspon.02G0014930 | 131.61 | 120.24 | 129.00 | 101.15 | 88.81 | 114.16 | 18.53 | 0.16 |
| Sspon.03G0007480 | 35.28 | 29.20 | 44.99 | 33.98 | 39.33 | 36.56 | 5.94 | 0.16 |
| Sspon.04G0035680 | 20.47 | 13.54 | 18.96 | 20.48 | 20.96 | 18.88 | 3.08 | 0.16 |
| Sspon.08G0011000 | 6.58 | 7.04 | 7.42 | 6.89 | 4.69 | 6.52 | 1.07 | 0.16 |
| Sspon.04G0006940 | 7.40 | 6.96 | 9.01 | 9.14 | 6.24 | 7.75 | 1.28 | 0.17 |
| Sspon.05G0027110 | 7.61 | 5.80 | 8.88 | 7.58 | 6.39 | 7.25 | 1.20 | 0.17 |
| Sspon.02G0048080 | 58.90 | 42.22 | 51.01 | 47.00 | 63.62 | 52.55 | 8.70 | 0.17 |
| Sspon.04G0024790 | 58.87 | 67.22 | 64.49 | 62.62 | 42.39 | 59.12 | 9.83 | 0.17 |
| Sspon.01G0003150 | 9.31 | 14.41 | 11.06 | 13.52 | 12.49 | 12.16 | 2.02 | 0.17 |
| Sspon.07G0005340 | 45.98 | 36.90 | 44.41 | 54.48 | 56.75 | 47.70 | 8.04 | 0.17 |

Note: The name and ID of these genes is from the Saccharum Genome Database.

(http://sugarcane.zhangjisenlab.cn/sgd/html/index.html )
