# Supplementary material for: Identification and evaluation of the novel genes for transcript normalization during female gametophyte development in sugarcane
Source: PeerJ. 2021 Oct 19;9:e12298. doi: 10.7717/peerj.12298 (PMC8532975; doi:10.7717/peerj.12298)
Supplement: Supplemental Information 3 [file peerj-09-12298-s003.docx]

**Table S3.** The qRT-PCR data and RPKM values for *CDK* and *REC8* in different developmental stages of sugarcane female gametophyte.

|  | RPKM values | | | | | |
| --- | --- | --- | --- | --- | --- | --- |
| Gene name | AC | MMC | Meiosis | Mitosis | Mature | SD |
| *CDK* | 27.17869 | 32.96066 | 29.24954 | 23.8338 | 14.35236 | 7.06 |
| *REC8* | 21.05071 | 22.10611 | 15.9815 | 21.47815 | 18.07605 | 2.61 |
|  | the average Ct values of qRT-PCR | | | | | |
| *CDK* | 34.24233 | 31.53731 | 30.41384 | 28.09803 | 32.43167 | 2.29 |
| *REC8* | 31.07758 | 28.47618 | 27.31267 | 27.15379 | 28.30417 | 1.57 |
