# Supplementary figures and images for: Identification and evaluation of the novel genes for transcript normalization during female gametophyte development in sugarcane
Source: PeerJ. 2021 Oct 19;9:e12298. doi: 10.7717/peerj.12298 (PMC8532975; doi:10.7717/peerj.12298)

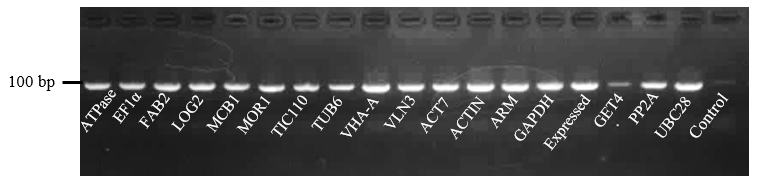

Supplement: Supplemental Information 4 [file peerj-09-12298-s004.png]

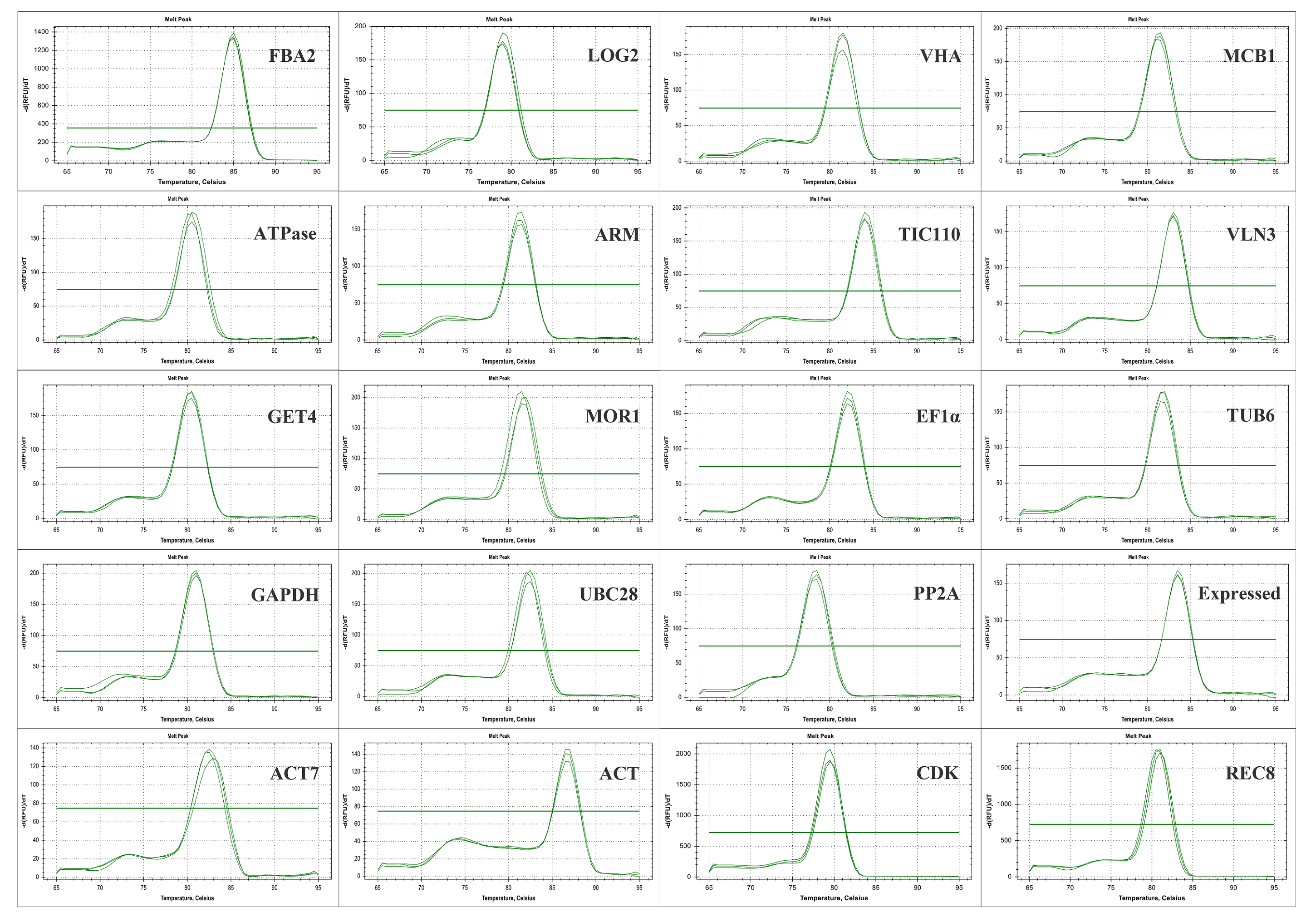

Supplement: Supplemental Information 5 [file peerj-09-12298-s005.png]

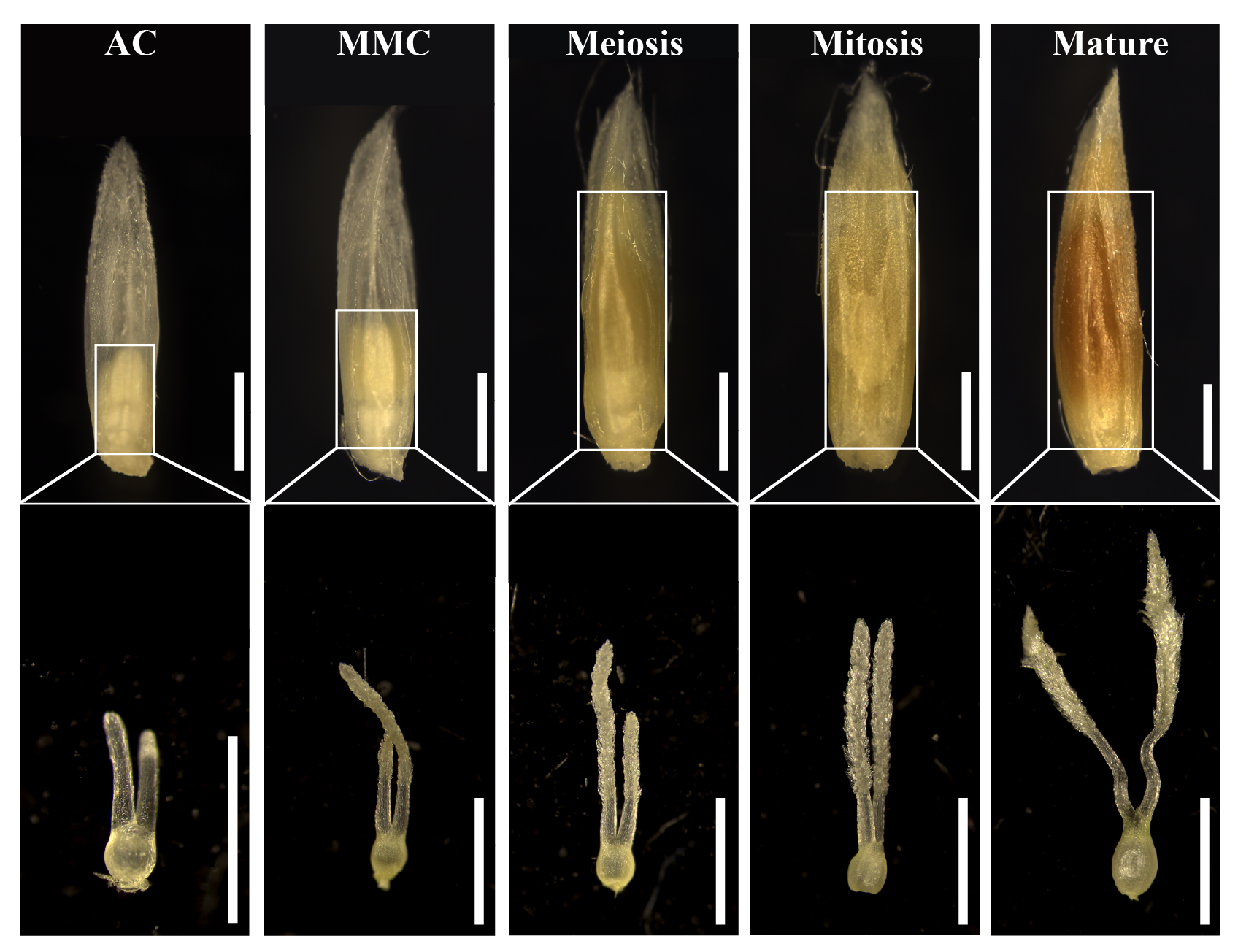

Supplement: Supplemental Information 6 [file peerj-09-12298-s006.png]
